# Reshaping of Bulbar Odor Response by Nasal Flow Rate in the Rat

**DOI:** 10.1371/journal.pone.0016445

**Published:** 2011-01-26

**Authors:** Emmanuelle Courtiol, Corine Amat, Marc Thévenet, Belkacem Messaoudi, Samuel Garcia, Nathalie Buonviso

**Affiliations:** Université Lyon 1, Centre National de la Recherche Scientifique, UMR 5020 Neurosciences Sensorielles, Comportement, Cognition, Lyon, France; Dalhousie University, Canada

## Abstract

**Background:**

The impact of respiratory dynamics on odor response has been poorly studied at the olfactory bulb level. However, it has been shown that sniffing in the behaving rodent is highly dynamic and varies both in frequency and flow rate. Bulbar odor response could vary with these sniffing parameter variations. Consequently, it is necessary to understand how nasal airflow can modify and shape odor response at the olfactory bulb level.

**Methodology and Principal Findings:**

To assess this question, we used a double cannulation and simulated nasal airflow protocol on anesthetized rats to uncouple nasal airflow from animal respiration. Both mitral/tufted cell extracellular unit activity and local field potentials (LFPs) were recorded. We found that airflow changes in the normal range were sufficient to substantially reorganize the response of the olfactory bulb. In particular, cellular odor-evoked activities, LFP oscillations and spike phase-locking to LFPs were strongly modified by nasal flow rate.

**Conclusion:**

Our results indicate the importance of reconsidering the notion of odor coding as odor response at the bulbar level is ceaselessly modified by respiratory dynamics.

## Introduction

Nasal airflow is the natural vector for odorant molecules so that respiration and odorant sampling are indissociable. Nasal airflow is thus a major parameter to take into consideration when studying olfactory processing in mammals, especially because sniffing parameters, such as frequency and flow rate (ml/min), are highly variable [1]–[3]. The importance of nasal airflow dynamics has been revealed at the olfactory epithelium (OE) level. First, olfactory receptor neurons (ORNs) have been shown to be sensitive to air pressure [4]. Second, low versus high flow rates differentially favor sorption of odorant molecules depending on their solubility [5]–[7], resulting in differential influence on activity patterns across the OE [8], [9].

Due to the anatomical organization of the epithelio-bulbar projections [10]–[12], a change in OE activity should be reflected in olfactory bulb (OB) activity. Indeed, optical recordings reveal that glomerular activation varies with sniffing frequency [13]–[15]or flow rate [16], and a sniff frequency-dependent attenuation of glomerular inputs has also been described [15]. At the level of individual mitral/tufted cell (M/T) activity, in the anesthetized rat, temporal patterns of M/T cells reflect the phasic stimulation of ORNs at each inspiration [17]. In the behaving rodent, natural changes in sniffing frequency lead to changes in the variability and spatial organization of M/T cell responses [18]. Importantly, temporal patterning of M/T cells is frequently lost during high frequency sniffing [18]–[20]. All of these studies have focused on the effects of sniffing frequency variation on bulbar activity. Conversely, the effects of nasal flow rate variation on M/T cell and local field potential (LFP) responses have not been studied, to our knowledge. It thus appeared essential to characterize nasal airflow-induced changes in OB response. If sampling behavior can change the characteristics of information carrier (spikes and LFP), then it would be necessary to reconsider the notion of odor coding. To investigate these modifications, we used a double cannulation and simulated nasal airflow protocol in anesthetized rats to uncouple nasal airflow from animal respiration. Both unit M/T cell extracellular activity and LFP were recorded during different conditions of nasal flow rate. We found that airflow changes in the normal range were sufficient to substantially reorganize the response of OB and determined that nasal airflow itself modifies spontaneous OB activity.

## Results

**Figure 1:**
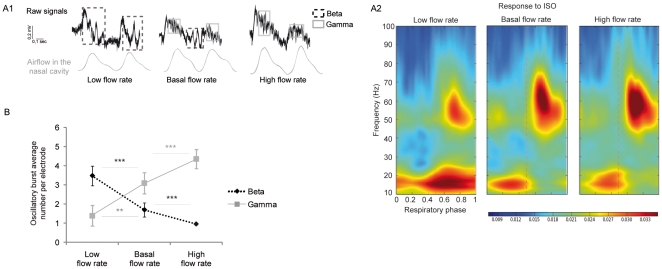
Modification of LFP oscillatory patterns. A1) Raw data collected from the same electrode in response to ISO under low (left), basal (middle) and high (right) flow rate conditions. A2) Representation of an average respiratory phase-frequency of LFP oscillatory activity under low, basal and high flow rate conditions in response to ISO. Amplitude is color-coded (the calibration scale below is common to the three representations in arbitrary units). Oscillation frequencies are represented relative to their position in the respiratory cycle, indicated in phase from 0 (beginning of inspiration) to 1 (end of expiration). Phase 0.5 indicates the transition between inspiration and expiration epochs. An average was calculated from signals recorded by all electrodes from 12 trials in each condition. B) Mean (± SEM) number of oscillatory bursts per electrode under low, basal and high flow rate conditions. For each trial, the same electrode was selected for the three flow rate conditions (n = 23 trials). Statistical test: Wilcoxon, *p<0.05, **p<0.01 and ***p<0.001.

Under basal flow rate conditions and for the majority of animals, we observed a temporal pattern of LFP signals in response to ISO similar to those of non-tracheotomized rats (Fig. 1A1 and A2, middle). The few animals in which we did not observe the alternation between beta and gamma oscillations under ISO stimulation at the basal flow rate were not retained for the analysis. Analyses were performed on 12 rats.

### Effect of nasal airflow on bulbar odor responses

Analyses were performed on 69 paired trials (23 trials for each flow rate condition) containing a total of 36 cells. To evaluate if nasal airflow shaped odor response at the olfactory bulb level, we first explored its influence on odor-induced LFP oscillations and then on M/T cell responses to odors. Next, we explored the influence of nasal airflow on the phase locking between M/T cell unit activity and LFP oscillations. For most analyses, since odors induced no statistical difference in the global proportion of respiratory-pattern activities, responses to odors were pooled.

#### LFP activity

Examples of typical signals in response to ISO are presented in Figures 1A1 and A2. In this example, while the classical alternation between gamma and beta oscillations on the respiratory cycle was observed at the basal flow rate (Fig. 1A1, A2, middle), low and high flow rate conditions induced LFP modifications. On the raw signal, the low flow rate (left) induced more numerous beta oscillatory bursts at the expense of gamma oscillatory bursts. In the example of Figure 1A2, the beta oscillatory burst appeared longer under low flow rate than under basal flow rate conditions. For analysis of the whole data set, we compared data from recordings obtained under low, basal and high flow rate conditions, performed at the same site from the same electrode under the three conditions. We first compared the number of beta or gamma oscillatory bursts per electrode and per recording under each flow rate condition (Fig. 1B). We observed that the mean number of gamma oscillatory bursts significantly increased with increasing flow rate (low versus basal: Wilcoxon  = 27.5, p<0.01; basal versus high: Wilcoxon  = 15, p<0.001). Conversely, the mean number of beta oscillatory bursts significantly decreased (low versus basal, Wilcoxon  = 7, p<0.001; basal versus high, Wilcoxon  = 17.5, p<0.001), indicating that nasal airflow can quantitatively change the LFP pattern. We next examined if the intrinsic characteristics of oscillations, such as duration, amplitude and frequency, could also be modified by flow rate. The only significant modifications we observed concerned gamma oscillations; the duration of gamma episodes was significantly decreased when flow rate was decreased relative to basal flow rate (Table 1, Wilcoxon  = 3, p<0.05), and their amplitude was significantly decreased (Table 1, Wilcoxon  = 3,p<0.05).

**Table 1:** Means (± SEM) of LFP intrinsic characteristics.

		Beta	Gamma
Characteristics	Flow rate	Average *(±SEM)*	Average *(±SEM)*
**Duration** (s)	Low	0.242 *(±0.016)*	0.101 *(±0.008)*
	Basal	0.209 *(±0.014)*	0.135 *(±0.011)*
	High	0.215 *(±0.163)*	0.132 *(±0.011)*
**Amplitude** (Arbitrary unit)	Low	9.137 *(±0.804)*	9.544 *(±0.677)*
	Basal	9.446 *(±0.846)*	12.479 *(±1.802)*
	High	8.548 *(±1.113)*	9.871 *(±1.134)*
**Frequency** (Hz)	Low	15.900 *(±0.462)*	52.821 *(±1.035)*
	Basal	16.949 *(±0.576)*	53.749 *(±1.273)*
	High	14.912 *(±0.560)*	54.656 *(±1.108)*

Duration (second), amplitude (arbitrary units) and frequency (hertz) are presented for beta and gamma oscillations and for the three flow rate conditions. Data from low and high flow rate conditions were compared to data from the basal flow rate condition (n = 23 trials). Statistical test: Wilcoxon, *p<0.05.

In summary, the occurrence probability for odor-evoked LFP oscillations was strongly modified by nasal flow rate, while the intrinsic characteristics of oscillations were less or not. The next step was to ask whether the M/T cell unit activity in response to odors was modified by nasal flow rate.

#### M/T cell activities

**Figure 2:**
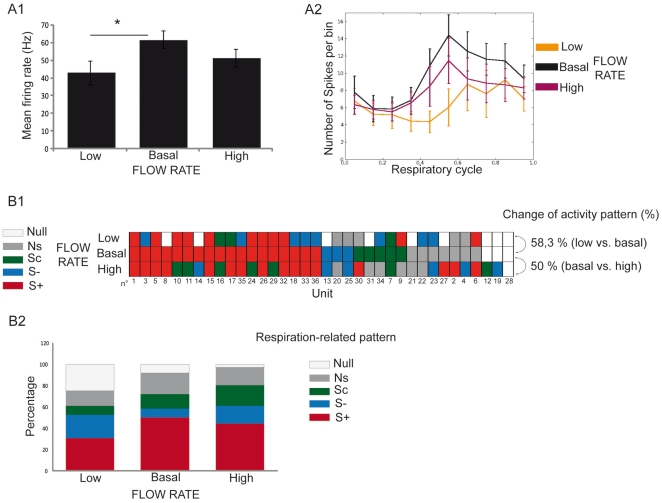
Nasal flow rate modifies mitral cell response to odors. Thirty six mitral cells were recorded under the three flow rate conditions. A1) Mean instantaneous frequency rate (± SEM) under each flow rate condition (n = 36). Paired t-test, *p<0.05. A2) Mean instantaneous frequency (or number of spikes per bin, ± SEM) as a function of respiratory cycle under the three flow rate conditions (low flow rate: orange, basal flow rate: black, and high flow rate: purple). B1) Matrix representing respiration-related patterns of each cell recorded under the three flow rate conditions. Each line represents a flow rate condition, and each column represents a unit. A color was attributed to each pattern: excitatory synchronized (S+, red), suppressive synchronized (S−, blue), complex synchronized (Sc, green), respiration non-related (NS, dark gray) and null activity (NULL, light gray). Cells are ordered according to their respiratory-related pattern at basal flow rate. The percent of change in activity pattern represents the percent of units exhibiting a different pattern under the two flow rate conditions. B2) Percentages of each activity pattern as a function of flow rate condition (n = 36). Low and high nasal flow rate conditions were compared to the basal flow rate. Statistical test: chi^2^.

We recorded 36 mitral cells under each flow rate condition. We first compared M/T cell responsiveness under the three flow rate conditions. A cell was considered responsive when its respiratory pattern and/or frequency changed from a spontaneous to an odor period. A decreasing flow rate resulted in a significant decrease in the percentage of responsive M/T cells (low: 66.7% vs. basal: 88.9%; Chi^2^ (1)  = 5.142, p<0.05). No significant difference was observed between basal and high flow rate conditions (88.9% vs. 91.7%, respectively; Chi^2^ (1)  = 0.158, p>0.05). When comparing the mean instantaneous firing frequency under the three flow rate conditions (Fig. 2A1), we observed that it reached approximately 50–60 Hz under the basal (61.58 Hz) and high (51.26 Hz) flow rate conditions, as reported in non-tracheotomized animal [21], [22]. Interestingly, it was significantly decreased under the low flow rate condition (low: 42.89 Hz vs. basal: 61.58 Hz). For a detailed view, see Figure S1 showing the matrix of the mean instantaneous frequency for each cell under each flow rate condition. We next compared the mean instantaneous frequency of M/T cells as a function of the respiratory cycle under each flow rate condition. Respiration-triggered histograms showed that M/T cell activity remained modulated by the respiratory rhythm, regardless of the flow rate (Fig. 2A2). Distribution of M/T cell mean instantaneous frequency relative to the respiratory cycle was compared using the Equal Kappa Test. This test revealed a significant difference of distribution between low and basal conditions (p<0.001) while there was no statistical difference between basal and high flow rate conditions.

We then compared the proportion of respiration-related patterns under the three flow rate conditions. Figure 2B1 shows the respiration-related patterns of a series of 36 M/T units recorded under the three flow rate conditions. Even though no significant difference appeared in the total proportion of each pattern between the three conditions (Fig. 2B2), 58.3 and 50% of the patterns were modified when airflow was decreased and increased, respectively, relative to the basal flow rate (Fig. 2B1). Thus M/T cell respiratory-pattern in response to the same odor is modified depending on imposed nasal flow rate.

We observed that nasal airflow influenced both OB unit and network activities. Finally, we investigated the temporal relationship between spikes and LFP oscillations.

#### Relation between LFP and unit activity

**Figure 3:**
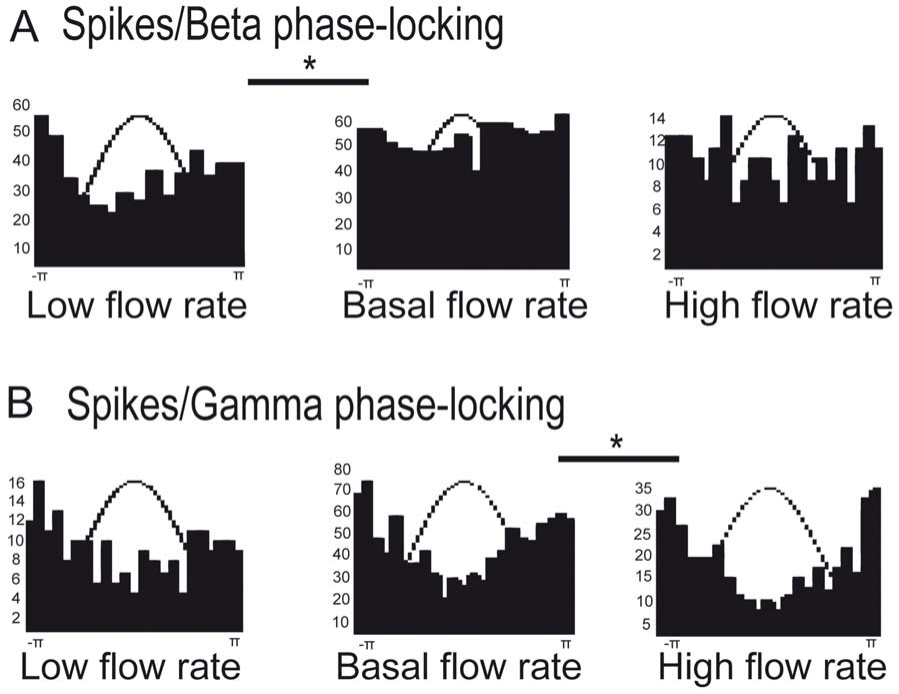
Spike phase locking to LFP oscillations is modified by nasal flow rate. Each histogram represents spike distributions relative to the oscillation phase for each flow rate condition. A) Phase locking between spikes and beta oscillations [number of spikes for each condition: low flow rate (n = 516), basal flow rate (n = 661) and high flow rate (n = 228)]. B) Phase locking between spikes and gamma oscillations [number of spikes for each condition: low flow rate (n = 188), basal flow rate (n = 1019) and high flow rate (n = 370)]. Statistical test: Equal Kappa test, *p<0.05.

To characterize the temporal relationships between oscillatory fields and individual spikes, we plotted the spike phase distribution for each cell for both beta and gamma bands under each flow rate condition. Phase histograms were then computed across cells. The significance of spikes/LFP phase locking was tested on the histograms (Rayleigh test; Fig. 3). Our analysis revealed a significant phase locking between spikes and beta oscillations only for the low flow rate condition. Conversely, a significant phase locking was observed between spikes and gamma oscillations for all three flow rate conditions (Rayleigh test, p<0.05). We then compared the strength of phase locking between the three flow rate conditions using the Equal Kappa test. This test revealed a significant difference of phase locking strength between basal and high flow rate conditions for the gamma oscillation (Fig. 3B). Hence, the higher the flow rate was, the stronger the spike phase locking to gamma phase was. Conversely, the lower the flow rate was, the stronger the spike phase locking to the beta phase was (Fig. 3A).

We showed here that changing flow rate modified odor response at the level of the OB. Since, in our experimental conditions, changing airflow modified both air pressure in the nasal cavity and the odorant stimulation (i.e., the quantity and migration of odor molecules), we then wanted to determine the relative influence of nasal airflow itself and that of the odor. Since odorant stimulation cannot be applied without nasal airflow, we attempted to answer this question by examining the effect of nasal airflow on OB spontaneous activity.

### Effect of deodorized nasal airflow on bulbar activity

First, we tested the effect of the presence of a respiration-modulated deodorized airflow (nasal airflow ON, 500 ml/min at the basal flow rate) versus its absence (nasal airflow OFF) in the nasal cavity on OB spontaneous activity (96 trials for each condition, with 45 cells detected). Effect of continuous deodorized nasal airflow on LFP activity was also tested on three additional rats. Second, we tested the effect of different nasal flow rates of deodorized air on M/T cell spontaneous activity (23 trials under low, basal and high flow rate conditions, with 36 cells detected).

#### LFP activity

**Figure 4:**
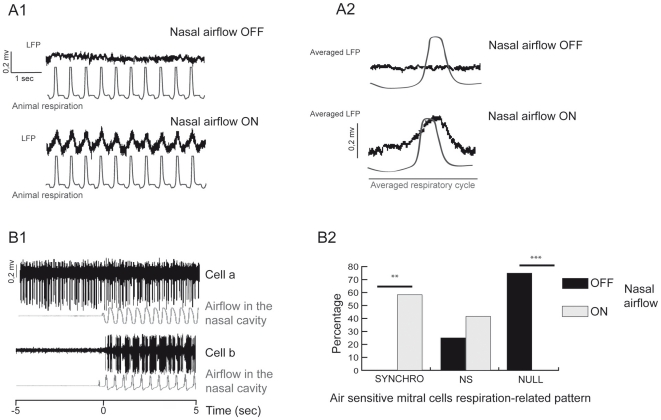
Nasal airflow (without any odor) imposes a respiration-related rhythm. A1) Dark traces: examples of field potentials recorded in the mitral cell layer when airflow was OFF (top) and ON (bottom). Gray traces: animal respiratory signal measured at the tracheal cannula. A2) Field potentials averaged over the respiratory cycle when airflow was OFF (top) and ON (bottom). B1) Forty five mitral cells were recorded under nasal airflow ON and OFF conditions. Raw data filtered at 300–3000 Hz of: an air-insensitive cell (a) and an air-sensitive cell (b). Bottom trace: airflow in the nasal cavity (500 ml/min) recorded at the nostril entrance. Nasal airflow was simulated at time 0. B2) Percentages of respiration-related (SYNCHRO), respiration non-related (NS) and null activity (NULL) patterns in air-sensitive cells when airflow was OFF (black) and ON (gray) in the nasal cavity (n = 12). Of these 12 cells, seven shifted from the NULL to SYNCHRO pattern, two from the NULL to NS and three from NS to NS (with a change in spike rate). Statistical test: Chi^2^, **p < 0.01, ***p<0.001.

Since there was no airflow through the nasal cavity in the OFF condition, we used animal respiration as the time base for signal analyses (Fig. 4A). Even though fast LFP oscillations did not appear without odor, the slow rhythm related to respiration (1–3 Hz) was present. As shown in Figures 4A1 and A2 (bottom), when deodorized airflow passed through the nasal cavity, we observed a slow rhythm. Conversely, under the nasal airflow OFF condition, we never observed the slow rhythm (Fig. 4A1, A2, top). We then tested the influence of flow rate modulation on spontaneous slow LFP activity. Surprisingly, no significant difference appeared in the slow LFP modulation amplitude when flow rate was decreased (200 ml/min) or increased (800 ml/min, data not shown). In addition, even by increasing or decreasing nasal flow rate, fast LFP oscillations did not appear. To complete the study of the effect of nasal airflow on slow LFP modulation activity, we tested the effect of a continuous nasal airflow at 200, 500 and 800 ml/min. On the 27 trials realized in three rats, we never observed a slow rhythm induced by a continuous nasal airflow whatever the flow rate condition (Figure S2).

#### M/T cell activity

Forty-five mitral cells were recorded both in nasal airflow ON and OFF conditions (deodorized air). When airflow was switched from OFF to ON (500 ml/min) in the nasal cavity, two M/T cell populations were revealed. First, we found air-insensitive M/T cells, defined as cells that did not change their temporal firing pattern and/or spike frequency when airflow was ON in the nasal cavity compared to the nasal airflow OFF condition. Out of 45 cells, 33 (73%) were insensitive to airflow. A representative example is shown in Figure 4B1 (a). Conversely, 12 cells (27%) were air-sensitive M/T cells and were characterized by a significant change in their spiking activity when airflow was ON in the nasal cavity [see Fig. 4B1 (b)]. This representative cell did not present activity when nasal airflow was OFF, but it presented respiration-related activity when nasal airflow was ON. To recapitulate pattern changes in air-sensitive mitral cells, respiration-related patterns were classified into three groups (Fig. 4B2): NULL, NS and SYNCHRO (in which S+, S-and Sc patterns were pooled). As shown in Figure 4B2, when nasal airflow was OFF, 75% of cells presented a NULL activity, and 25% presented an NS activity. When nasal airflow was turned ON, the majority of air-sensitive cells adopted an activity synchronized with respiration (SYNCHRO: 58.33%), whereas NULL activity no longer existed.

**Figure 5:**
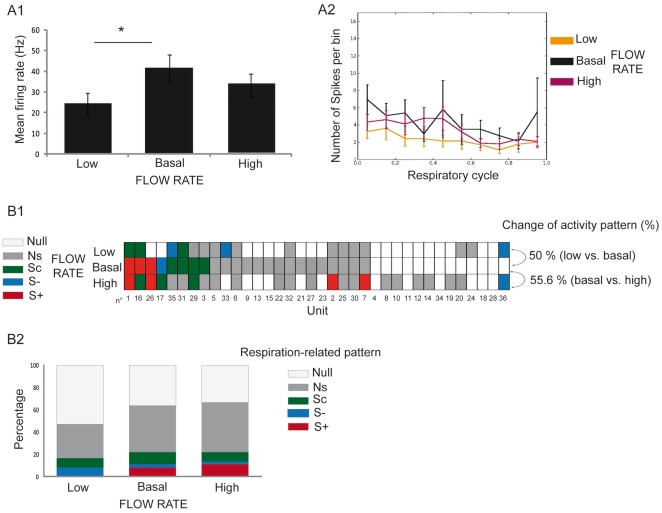
Spontaneous activity of OB units is modified by nasal flow rate. Thirty six mitral cells were recorded under the three flow rate conditions. A1) Mean instantaneous frequency rate (± SEM) under each flow rate condition (n = 36). Paired t-test, *p<0.05. A2) Mean instantaneous frequency (± SEM) as a function of respiratory cycle under the three flow rate conditions (low flow rate: orange, basal flow rate: black, and high flow rate: purple). B1) Matrix representing respiration-related spontaneous patterns of each cell recorded under the three flow rate conditions. Each line represents a flow rate condition, and each column represents a unit. A color was attributed to each pattern: excitatory synchronized (S+, red), suppressive synchronized (S−, blue), complex synchronized (Sc, green), respiration non-related (NS, gray) and null activity (NULL, light gray). Cells are ordered according to their respiratory-related pattern at basal flow rate. The percent of change in activity pattern represents the percent of units exhibiting a different pattern under the two flow rate conditions B2). Percentages of each spontaneous activity pattern as a function of flow rate condition (n = 36). Low and high nasal flow rate conditions were compared to the basal flow rate. Statistical test: Chi^2^.

Mitral cell spontaneous activity was then studied under the three flow rate conditions. A total of 36 cells were recorded under each flow rate condition (deodorized air). When comparing the mean instantaneous frequency rate between flow rate conditions (Fig. 5A1), we observed, as in response to odors, that spontaneous activity had a lower mean instantaneous frequency rate in the low flow rate condition (basal: 41.15 Hz vs. low: 24.06 Hz; p<0.05). No significant difference was observed between basal and high flow rate conditions. For a detailed view, see Figure S1 showing the matrix of the mean instantaneous firing frequency for each cell under each flow rate condition. We also compared the mean instantaneous frequency as a function of respiratory cycle for each flow rate condition (Fig. 5A2). No obvious modulation of the instantaneous frequency was present, with the exception of a slight one at the high flow rate. Equal Kappa test did not reveal any significant difference of instantaneous frequency distribution relative to the respiratory cycle between the three flow rate conditions.

To explore to what extent M/T cell activity pattern modifications in response to odors were modified by nasal airflow variation, we next studied the effect of nasal flow rate modulation on the spontaneous activity patterns of M/T cells in the absence of any odor (Fig. 5B1). In a global view, proportions of the spontaneous activity respiratory patterns differed from those under odor conditions, as the NULL and NS patterns were the patterns most observed regardless of the flow rate condition. As seen in response to odors, even though no specific activity pattern appeared when flow rate was increased or when it was decreased (Fig. 5B1, B2), 50 and 55.6% of these patterns were modified when airflow was decreased and increased, respectively (Fig. 5B1).

## Discussion

The aim of this study was to examine the extent that OB odor response was influenced by nasal flow rate variation. For this purpose, we used a double tracheotomy paradigm coupled with a respiratory cycle simulation in the nasal cavity. We extended and corroborated the earlier conclusion of various authors [4], [23], [24] showing that nasal airflow itself imposes a respiratory rhythm to OB activity. Importantly, we observed that flow rate modifications induced variations of different odor information carriers: LFP oscillatory activity, M/T single cell activity and spike phase locking to LFP oscillations.

### Nasal airflow tunes OB activity during the respiratory cycle

Adrian [23] first demonstrated a periodic LFP activity in the OB related to the animal's respiration. On unitary level, M/T cells fire in relation with air intake in absence of olfactory stimuli [25], [26]. Similarly, olfactory receptor neuron terminals are activated by natural sniffing of deodorized air in the awake rat [24]. Recently, Grosmaitre [4] proposed an explanation about respiration-related rhythmicity by showing that ORNs are sensitive to air pressure. Consistent with these results, we showed that airflow suppression in the nasal cavity led to suppression of respiration-related slow LFP oscillation (Fig. 4A). Application of a continuous nasal airflow did not led to respiration-related slow LFP oscillation whatever the flow rate (Figure S2). At mitral cell activity level, a continuous nasal airflow did not induce a respiration related pattern in mitral cells; conversely, mitral cells adopted a continuous firing [17]. Effect of central structures as a source of respiratory modulation could not be excluded [27]. Periphery and central structures probably act in concert. However, in our conditions, nasal airflow seems to be the most prominent source of respiratory modulation.

Second, we observed a population of M/T cells that adopted a respiration-related pattern of activity when airflow was applied to the nasal cavity (Fig. 4B). These observations appear to confirm the hypothesis according to which mechanosensitivity of ORNs may be a peripheral drive to synchronize OB rhythmic activity with respiration [4]. This respiratory tuning at the level of the network and M/T cells constitutes a major process for synchronizing OB and higher order structures, such as the piriform cortex, which also presents respiration-related activity [28]–[30]. Bulbar respiratory tuning by nasal airflow could permit an optimal coupling between olfactory structures, which may be important for information transmission. Furthermore, this bulbar “pre-activation” by nasal airflow could prepare the OB to process odor stimuli [31]. By imposing a basal activity to the OB, nasal airflow may provide an excitatory source to the OB, which in turn could facilitate its response to odors.

### Nasal flow rate strongly impacts odor response in the OB

Numerous authors have shown that OE odor responses are modified by nasal flow rate [7]–[9]. Such modifications have also been revealed at the glomerular level. In particular, it has been recently reported that glomerular responses are affected by nasal flow rate [16]. Considering this, our data show for the first time that the response to odors of both OB network and output (M/T cells) are modified by nasal flow rate variations (Fig. 1 and Fig. 2). We showed that modifications of nasal airflow not only influence bulbar input but also its output activity. At the network level, gamma oscillations appeared more often under the high nasal flow rate, while beta oscillations appeared more often under the low flow rate. These results can be simply interpreted in terms of bulbar activation related to the odorant stimulation level which can be induced by each flow rate. Gamma oscillation prevalence is likely due to the high bulbar activation related to a high flow rate. It has been shown that the gamma oscillation is favored by high odor concentration [32] and high vapor-pressure molecules [33]. Conversely, beta oscillation prevalence is likely due to poor bulbar activation (related to a low flow rate), as it has been shown that the beta oscillation is favored by low odor concentrations [32] and low vapor-pressure molecules [33]. Similarly, at the M/T cell level, changes in odor responsiveness as a function of nasal flow rate may also be attributed to the level of bulbar activation. Finally, we observed that spike phase locking to LFP oscillations was also modified as a function of the flow rate condition (Fig. 3). A high flow rate improved spike phase locking to gamma, whereas a low flow rate improved spike phase locking to beta. Different authors have suggested that spike phase locking to LFP oscillations is a key parameter in olfactory coding, at least in the honeybee (for review, see [34]) and fish [35]. If, as we report, an odorant can evoke different phase locking and LFP oscillatory patterns according to nasal flow rate and the animal's sniffing dynamics, then such hypotheses should be reformulated.

Our finding that OB activity is modified by nasal airflow variation raises a new question: Do the observed modifications simply reflect the differential ORN activation due to air pressure variation, or are they due to variations in odorant stimulation because of flow rate variations? Unfortunately, odors cannot be delivered without airflow into the nasal cavity, making it impossible to directly answer this question. Nevertheless, some clues can be found in our data. First, we observed that M/T cells, in which the response to odors changed with flow rate, were not systematically those cells whose spontaneous activity was modified by flow rate (Figure S1). Second, slow LFP modulation was not modified by flow rate when no odor was delivered. Third, even with flow rate modification, gamma or beta oscillations were never evoked without odor. It thus seems reasonable to assume that the modifications in odor response we observed were not the only consequence of air pressure variation, but rather such modifications are likely due to both variations in nasal airflow pressure and odorant stimulation. If nasal flow rate variation affects odorant stimulation, it can do it in different ways: first by changing odorant concentration and second by modifying odorant migration through the nasal cavity. Further studies will next be required to specify the respective implication of each of these parameters by using both a systematic panel of odorant concentrations and a panel of odorants with different physico-chemical properties.

### Functional implications for odor perception

By demonstrating that nasal flow rate induced modifications in bulbar network activity, M/T single cell activity and spike phase locking to LFP, we have presented novel evidence showing that nasal airflow is a key parameter to consider when studying olfactory coding. This result leads to two questions. First, what allows perception stability? Indeed, it has been recently shown that even though glomerular maps are modified by odorant concentration, animal odor perception remains stable [36]. An interesting explanation can be found in Bathellier et al. [37]. They showed that relevant information for odor coding is contained in mitral cell ensemble activity which is robust to changes in sniffing frequency. We could consider the possibility that such a coding scheme should also be robust to changes in flow rate variations.

Second, what is the role of respiratory dynamics in perception? Different authors have considered respiratory dynamics not only as the odorant molecules vector but also as an integral part of the olfactory percept [38], [39]. As demonstrated in humans, there are fast adjustments of sniff volume depending on odorant concentration [40]. These fast modulations of respiratory dynamics suggest that olfactomotor control could be similar to that of vision or audition [40]. Variations in sniff parameters would serve to optimize the transport of odorant molecules along the OE pathway [41], [42], similar to how eye movements serve to allow acute visual perception [43].

## Materials and Methods

### Preparation and recording

Male Wistar rats (200–450 g) obtained from Janvier (Le Genest-Saint-Isle, France) were anesthetized with urethane (1.5 mg/kg, i.p., with additional supplements as needed) and placed in a stereotaxic apparatus. LFP oscillations were used to monitor anesthesia depth. Animals were placed on a heating pad to maintain constant body temperature.

#### Ethics Statement

All surgical procedures were conducted in strict accordance with the European Community Council directive of November 24, 1986 (86/609/EEC), those of the French Ethical Committee and French Legislation and received approval from the Lyon 1 University Ethics Committee (Direction of veterinary service # 69387473).

#### Tracheotomy

**Figure 6:**
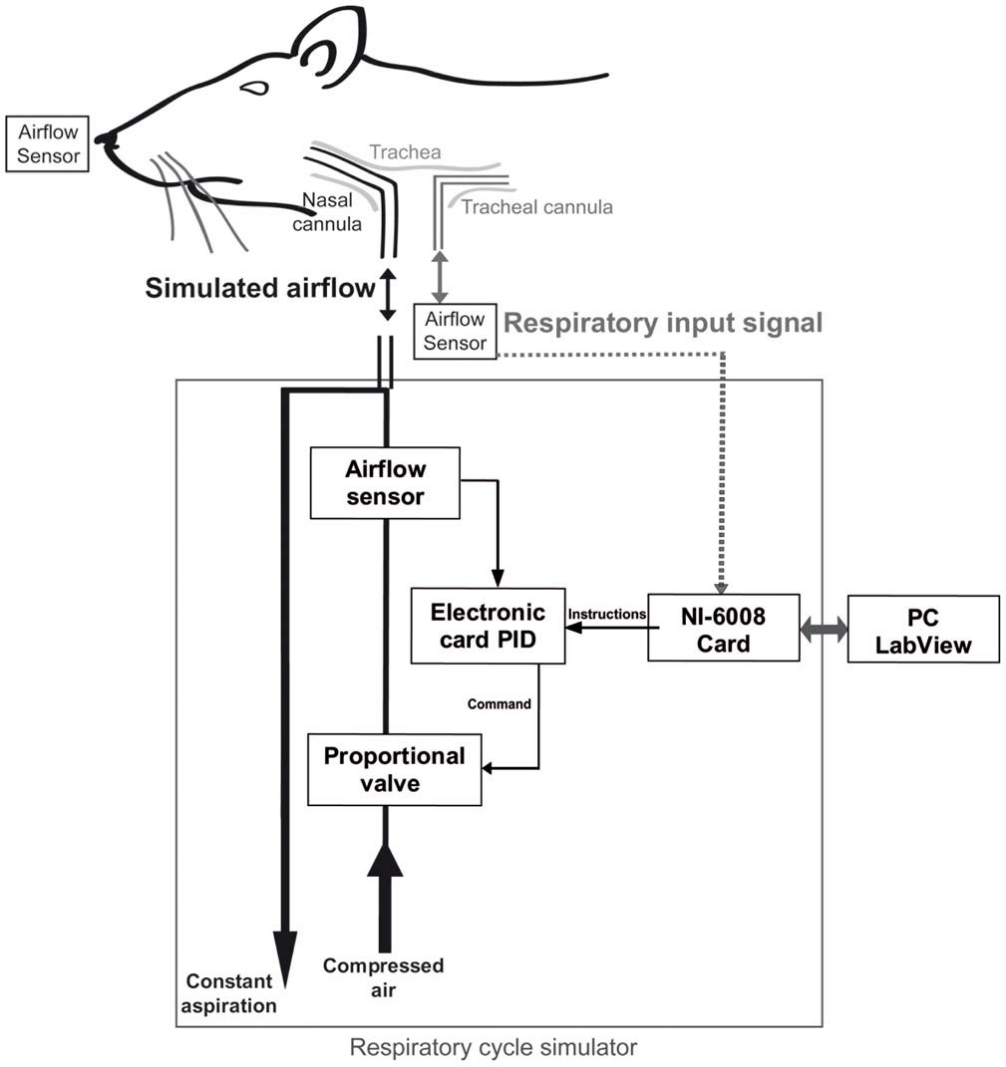
Respiratory cycle simulator. This apparatus consists of an electronic part (PID card, airflow sensor and proportional valve), an acquisition card (NI-6008 analog input/output) and a PC (software under LabView®). Using a constant aspiration and a compressed air input passing through the system allowed the simulation of both inhalation and exhalation phases of the respiratory cycle. The simulated airflow was directly sent to the nasal cavity via a nasal cannula. The tracheal cannula allowed the animal to freely breathe and was used to acquire the animal's respiratory signal.

Once all pain reflexes were abolished, a tracheotomy was performed by inserting a first cannula into the trachea, which allowed the rat to breathe freely (catheter Biotrol, int. 1.57 mm, ext. 2.08 mm, tracheal cannula in Fig. 6). A second cannula was then inserted rostrally through the larynx to the postnasal cavity to allow air to be pushed and pulled through the nasal cavity (catheter Vygon, Venolux 247, int. 0.8 mm, nasal cannula in Fig. 6).

#### Electrophysiological recordings

The dorsal region of the OB was exposed. Bulbar activity was recorded as a broadband signal (0.1 Hz to 5 kHz) using 16-channel silicon probes (NeuroNexus Technologies, Ann Arbor, MI) with a homemade 16-channel DC amplifier. Data were digitally sampled at 10 kHz and acquired with a PC using an IOTech acquisition system (Wavebook, IOTech Inc., Cleveland, OH). Probes were placed in the lateral or medial part of the OB at such a depth that the maximum number of channels could be located within, or close to, the mitral cell layer. The mitral cell layer was located by a set of criteria: LFP waveform, magnitude of unit action potentials and the inability to record spikes from the granule cell layer. Recordings were performed in the whole antero-posterior axis of the OB.

#### Odors

Odors (Sigma Aldrich, Fluka) were delivered in a randomized series through a dilution olfactometer (400 ml/min). The odors were isoamyl acetate (ISO), 2-heptanone (K07), 1-decanol (A10) and p-Cymen (CYM). We chose to stimulate animals with several odors in order to increase the probability of cell responses. All odors were delivered in front of the animal's nose at a fraction of 18.10^−2^ of the saturated vapor pressure. The time delay between each odor presentation was at least 1 min. The recording protocol was as follows: 5 s of spontaneous activity, 5 s of odor-evoked activity and 5 s of post-stimulus activity.

#### Protocols for simulated airflow variations

Airflow was measured by fast response time airflow sensors (bidirectional micro bridge mass airflow sensor, AWM 2000 series, Honeywell®). This setup is extensively described in [44]. We used two sensors (see Fig. 6), one placed in front of the tracheal cannula to measure the animal's respiration and another placed at the entry of the nostril to measure airflow circulating through the nasal cavity.

To simulate respiratory cycles, we used a homemade apparatus that allows the reproduction of both inhalation and exhalation phases. The animal's respiratory signal (collected at the tracheal cannula) was sent to the respiratory signal simulator, which in turn sent a simulated airflow toward the nasal cavity through the nasal cannula (see Fig. 6). Imposed airflow through the nasal cavity was deodorized by passing through an activated carbon filter device (Carbon-Cap ^tm^ 75, Whatman filter, Bioblock). The whole system was based on a proportional-integral-derivative (PID) controller composed of an electronic card, a miniature proportional valve (VSO® model, Parker Precision Fluidics) and an air flow sensor (micro bridge mass airflow sensor, AWM 40000 series, Honeywell®). The electronic card was controlled via an analog data acquisition card (National Instruments® NI-USB 6000 series). This card was connected to a PC, and software developed under LabView® was designed to perform several tasks. The user was able to choose the simulated airflow parameters (time course and rate). To maintain conditions as physiological as possible, the simulated airflow was synchronized to the tracheal respiratory signal. In some cases, animal respiration was too irregular to permit the device to keep the synchrony between both signals (i.e., the simulated nasal airflow and animal respiration). We thus discarded trials in which both signals were not synchronized. To estimate the synchronization between simulated nasal airflow and animal respiration, we measured the delay between both signals at the inspiration/expiration transition (I/E) points. We considered both signals to be synchronized when the shift was <10% of the respiratory cycle. In selected trials, the shift was an average 4.63% (±3.12%) of the respiratory cycle.

Since the aim of this work was to study the influence of airflow variation, we chose to impose three different nasal airflow rates: low (200 ml/min), basal (500 ml/min) and high (800 ml/min) without varying any other parameter of the respiratory signal. These values refer to the maximum flow rate for the respiratory cycle. A 500 ml/min flow rate was chosen as basal because it reproduced the bulbar LFP signal in response to ISO that is typically recorded under the anesthetized non-tracheotomized condition [21]. This basal flow rate was adjusted to 500 ml/min ±50 ml/min depending on the animal state. The three flow rates employed corresponded to the physiological scale in rat measured in behaving animals [1]. To determine how nasal airflow itself could affect olfactory bulb activity, we tested the effect of nasal airflow presence (nasal airflow ON, 500 ml/min, without any odor, modulated at animal respiratory frequency) and absence (nasal airflow OFF) on olfactory bulb activity. In all of these trials, airflow was discontinuous and exactly reproduced animal's breathing frequency. To ensure us that imposed airflow in the nasal cavity was completely deodorized, we included a blank trial in each recording session. We never observed any bulbar response (neither LFP nor mitral cell activity) to the blank. We also tested, on 3 additional rats, the effect of a continuous nasal airflow at 200, 500 or 800 ml/min.

### Data processing

All data processing was performed using Openelectrophy open-access homemade software [45].

#### Respiratory signal

An important feature of the olfactory signal is its temporal correlation with breathing. We developed a method to represent data as a function of the respiratory phase [44]. Briefly, the recorded respiratory signal was processed to extract each respiratory epoch. The time component of these periods was then converted into a circular phase component defined between 0 and 1, which represented the beginning of the inspiration and end of the expiration, respectively. As a result, electrophysiological signals were no longer represented as a function of time but as a function of respiratory phase. The main advantage of this method is that the phase representation was common to all trials, as opposed to time representation. Electrophysiological recordings were analyzed relative to the respiratory cycle and in particular to the transition points between inhalation and exhalation (I/E). I/E points were automatically detected as zero-crossings of the respiratory signal, corresponding to the point of null airflow of the rising phase.

#### LFPs

Wavelet transform LFPs were obtained by band-pass filtering the recorded signal at 5–200 Hz. To preserve both time and frequency information, we used a time-frequency representation based on the continuous wavelet transform method.

Wavelet ridge extraction: We previously developed an algorithm [46] to extract phase information from the identified oscillations in the signal. Briefly, for each frequency band of interest, we computed the mean and standard deviation (SD) of the time-frequency map and defined the threshold as the mean +5 SDs of the time-frequency amplitude contained in the pre-stimulus period (between 0 and 5 s). These thresholds were used to define time and frequency boxes centered on points of maximum amplitude in the signal, bounded by small time and frequency ranges. Finally, we ran a high resolution Morlet's complex wavelet transformation on each box. Ridges were extracted on a Morlet scalogram time frequency map. Each time frequency ridge line represented all parameters of one oscillation (phase, frequency, amplitude, starting and ending times) as a function of time.

#### Spikes

Spike sorting: Signals from individual electrodes were amplified (gain 1000×) and filtered from 300 to 5000 Hz. Multi-unit activity consisted of a few neurons on each electrode. We chose to use only the well-discriminated units (with a signal-to-noise ratio ≥5:1) and to sort cells according to their spike amplitudes. We verified that all sorted cells exhibited a minimal 4 ms refractory period. Consequently, the number of units retained for analysis was restricted to 1–3 units per channel. We preferred to use a very strict procedure, which resulted in a limited number of units but was also very safe. With this conservative procedure, we were very confident in the quality of the sorting; all units were well isolated, and there were no duplicates.

Respiratory patterns: As previously described [47], M/T cell activity is well characterized as a function of its temporal pattern along the respiratory cycle. To evaluate such patterns, the time occurrence of each spike was converted into a respiratory phase (0–1). All data were plotted as histograms (divided into 20 bins) that represented the spike rate along the respiratory cycle. Histograms were classified into different types based on the classification described in [47]. For the present study, we reduced this classification to four types: i) *non-synchronized patterns (NS)*, characterized by a uniform distribution of spiking activity along the respiratory cycle; ii) *excitatory-simple-synchronized patterns (S+)*, presenting a single increase in firing activity along the respiratory cycle; iii) *suppressive-simple-synchronized patterns (S−)*, presenting a single decrease or stop in firing activity along the respiratory cycle; and iv) *complex-synchronized patterns (Sc)*, exhibiting multiple firing frequency changes along the respiratory cycle. A period with no or very few spikes during the considered epoch was classified as *NULL*.

Spike-LFP phase coupling. Our wavelet ridge-based analysis method of LFPs allowed an accurate estimation of the oscillation phase. An absolute phase was assigned to each action potential that occurred during an oscillatory epoch. The mean distribution of action potentials relative to the phase of the LFP oscillation (beta or gamma) was represented by phase histograms (23 bins), where the peak and trough of the wave were assigned to 0 and pi, respectively. From these histograms, circular mean, deviation and dispersion were calculated.

#### Statistics

Statistical tests were performed using Excel, Statview software or R combined with Python script. The level of significance was set at p<0.05 for all statistical tests (p<0.05 *, p<0.01 ** and p<0.001 ***).

LFP: LFP spontaneous slow modulation was calculated from the difference between the maximum and minimum points on each averaged LFP signal relative to the respiratory cycle. For fast LFP oscillations, average duration, frequency, amplitude and number of oscillatory bursts (defined as the mean number of detected oscillatory bursts per electrode under each flow rate condition) in the OB were compared between flow rate conditions using the Wilcoxon paired test. For all analyses, oscillation characteristics at basal flow rate were taken as the reference point for comparisons.

Spikes: First, M/T cell instantaneous frequency discharges were compared between the three flow rate conditions using a paired t-test. For each cell, instantaneous frequency discharge at the basal flow rate was taken as the reference point for comparisons. Second, distribution of M/T cell instantaneous frequency along the respiratory cycle was compared between the three flow rate conditions using the Equal Kappa test. Third, the probability of M/T cell respiration-related patterns were compared between flow rate conditions during spontaneous and odor-evoked activities using a Chi^2^ test. M/T cell activity patterns were also compared in nasal airflow ON vs. OFF conditions using a Chi^2^ test.

Spikes-LFP phase coupling: Rayleigh's uniformity test was used to calculate the probability that the spikes were uniformly distributed throughout the entire duration of an oscillatory cycle (null hypothesis). The circular Equal Kappa test was used to test the difference in spike distribution relative to oscillation cycle between the nasal flow rate conditions.

## Supporting Information

Figure S1 **Matrices of spontaneous and odor-evoked activities of OB units.** A) Matrix representing respiration-related spontaneous and odor-evoked patterns of each cell recorded under the three flow rate conditions. Each line represents a flow rate condition, and each column represents a unit. A color was attributed to each pattern: excitatory synchronized (S+, red), suppressive synchronized (S-, blue), complex synchronized (Sc, green), respiration non-related (NS, gray) and null activity (NULL, light gray), ordered by cell. B) Matrix representing spontaneous and odor-evoked instantaneous frequency of each cell recorded under the three flow rate conditions. Each line represents a flow rate condition, and each column represents a unit. Gray scale was used to represent firing rate from <20 Hz to >80Hz, ordered by cell.
(TIF)

Figure S2 **Continuous nasal airflow does not induce respiratory modulation.** Example of LFP signal recorded in different airflow conditions from left to right: nasal airflow OFF, continuous 200 ml/min, continuous 500 ml/min and continuous 800 ml/min. LFP signals are averaged over the respiratory cycle. Gray traces: averaged respiratory cycle measured at the tracheal cannula.
(TIF)
