# Supplementary figures and images for: Reshaping of Bulbar Odor Response by Nasal Flow Rate in the Rat
Source: PLoS One. 2011 Jan 26;6(1):e16445. doi: 10.1371/journal.pone.0016445 (PMC3027679; doi:10.1371/journal.pone.0016445)

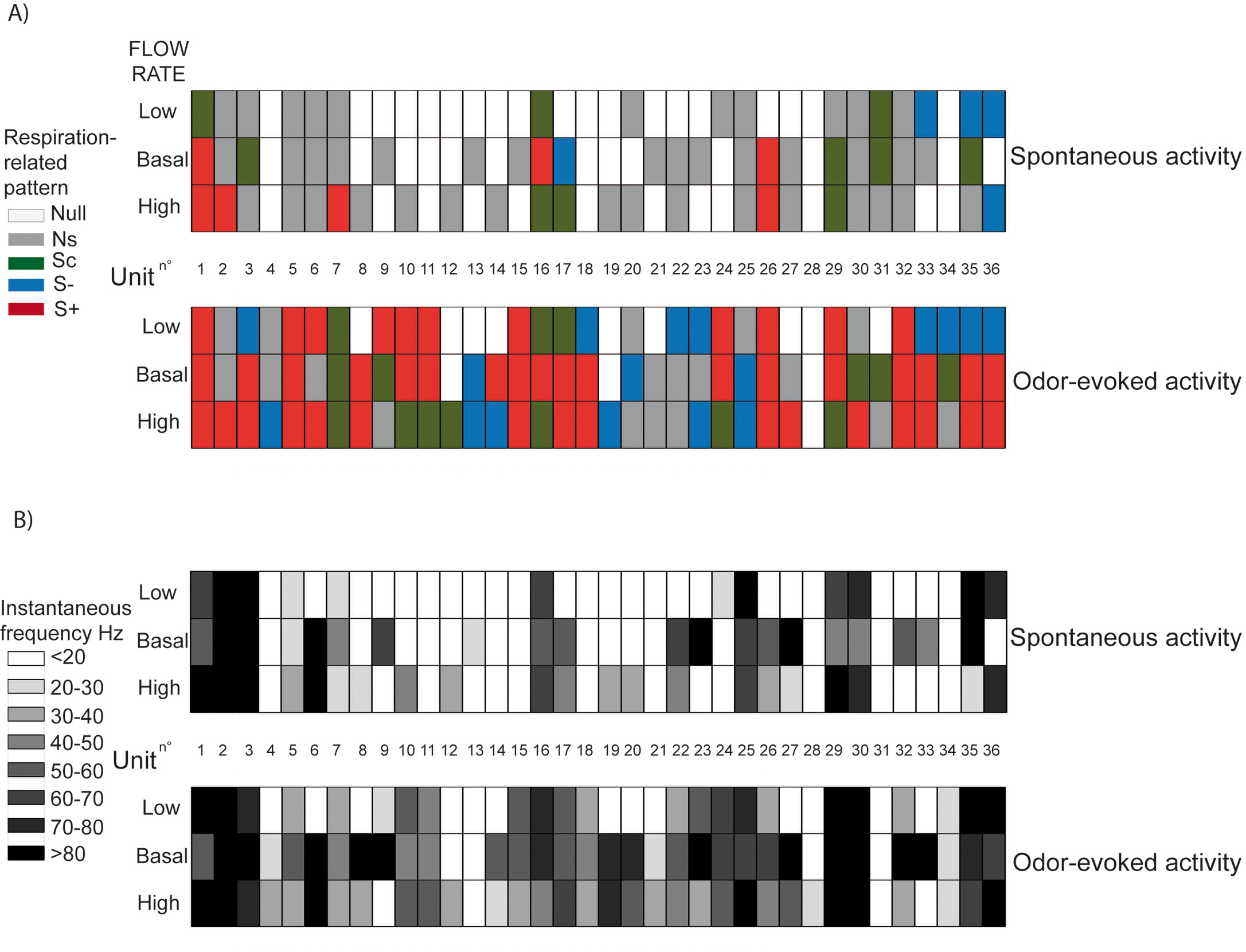

Supplement: Figure S1 — Matrices of spontaneous and odor-evoked activities of OB units. A) Matrix representing respiration-related spontaneous and odor-evoked patterns of each cell recorded under the three flow rate conditions. Each line represents a flow rate condition, and each column represents a unit. A color was attributed to each pattern: excitatory synchronized (S+, red), suppressive synchronized (S-, blue), complex synchronized (Sc, green), respiration non-related (NS, gray) and null activity (NULL, light gray), ordered by cell. B) Matrix representing spontaneous and odor-evoked instantaneous frequency of each cell recorded under the three flow rate conditions. Each line represents a flow rate condition, and each column represents a unit. Gray scale was used to represent firing rate from <20 Hz to >80Hz, ordered by cell. (TIF) [file pone.0016445.s001.tif]

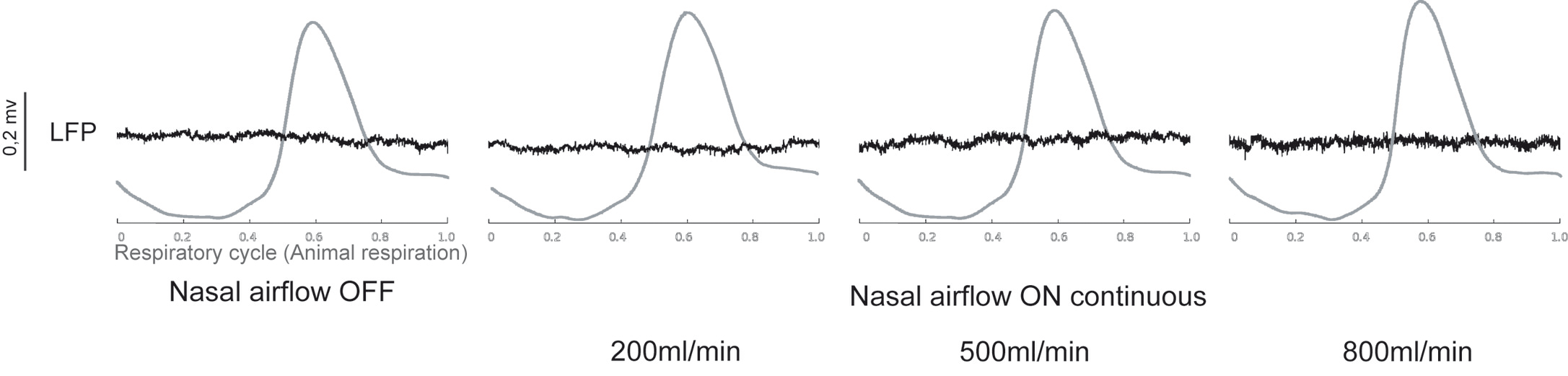

Supplement: Figure S2 — Continuous nasal airflow does not induce respiratory modulation. Example of LFP signal recorded in different airflow conditions from left to right: nasal airflow OFF, continuous 200 ml/min, continuous 500 ml/min and continuous 800 ml/min. LFP signals are averaged over the respiratory cycle. Gray traces: averaged respiratory cycle measured at the tracheal cannula. (TIF) [file pone.0016445.s003.tif]
